# Preparation of triangular silver nanoparticles and their biological effects in the treatment of ovarian cancer

**DOI:** 10.1186/s13048-022-01056-3

**Published:** 2022-11-21

**Authors:** Man Yin, Xiangyu Xu, Hui Han, Jiahui Dai, Ronghe Sun, Linqing Yang, Junyu Xie, Yunfei Wang

**Affiliations:** 1 Department of Clinical Medicine, Jining Medical University, Jining, 272000 Shandong China; 2 Laboratory of New Antitumor Drug Molecular Design & Synthesis, College of Basic Medical, Jining Medical University, Jining, 272067 Shandong Province China; 3 Department of Gynecology, Affiliated Hospital of Jining Medical University, Gu Huai Road, No.89 Jining, 272029 Shandong China

**Keywords:** Silver nanoparticles, Ovarian cancer, Antitumor activity, Nanotargeted therapy

## Abstract

**Background:**

In recent years, silver nanoparticles (AgNPs) have gradually been widely used, especially in the field of anticancer medicine. Ovarian cancer (OC) is the gynaecological malignancy with the highest mortality rate, and the current treatment is still based on surgery, chemotherapy and postoperative targeted therapy. Therefore, the development of safe and effective nanoparticles for targeted therapy of OC is very important. This study aimed to prepare a new type of triangular silver nanoparticles (tAgNPs) and evaluate the anticancer properties for OC in vitro and in vivo.

**Methods:**

The tAgNPs were chemically synthesized and characterized using scanning electron microscopy (SEM), ultraviolet (UV) spectrophotometry and other techniques. By performing cell-based tests, such as cell counting kit-8 (CCK-8), plate colony formation, cell apoptosis, reactive oxygen species (ROS), and western blot (WB) assays, the inhibitory effects and related mechanisms of tAgNPs on OC cells were analysed.The anticancer effect of tAgNPs in vivo was verified by a SKOV3 tumor-bearing mouse model.

**Results:**

Five types of tAgNPs with different colours were successfully synthesized, with a particle size of 25–50 nm and a good dispersion. The results of in vitro experiments showed that tAgNPs treatment reduced the viability and proliferation of SKOV3 cells, arrested the cell cycle in G0/G1 phase, inhibited the expression levels of proliferation-related factors and cyclins, and promoted cell apoptosis by producing ROS and increasing caspase-3 activity. Consistent with the results of in vitro experiments, in vivo animal experiments also showed that tAgNPs significantly inhibited the proliferation of ovarian cancer. More importantly, no obvious toxic and side effects were observed.

**Conclusions:**

In this study, a novel triangular AgNPs was successfully prepared. tAgNPs are very stable, significantly inhibit the proliferation of OC cells and tumour growth in tumour-bearing mice, providing a promising nanotargeted therapy for OC.

**Supplementary Information:**

The online version contains supplementary material available at 10.1186/s13048-022-01056-3.

## Introduction

Ovarian cancer (OC) is one of the three most common malignant tumours of the female reproductive system and one of the most common causes of death among gynaecological malignancies [1]. Currently, effective screening strategies for OC are still lacking; therefore, approximately 70% of patients are diagnosed with OC when they are already in the late stage of the disease and have lost the optimal time for treatment [2–4] . A variety of treatments have been developed for OC, such as surgery, chemotherapy, radiotherapy, immunotherapy, and targeted therapy. Among them, surgery and chemotherapy are still the main treatments. On the one hand, chemotherapy has side effects, such as damage to normal cells and gastrointestinal reactions; on the other hand, drug resistance may occur in patients receiving chemotherapy, leading to a high recurrence rate [5] . Overall, the prognosis of patients with OC is poor. Therefore, the identification and development of new, effective and harmless treatments is very important.

Nanoparticles have a large surface area and volume ratio. Due to this unique characteristic, nanoparticles have been widely used in the fields of industry, textiles, biosensors, biotechnology, and medicine [6, 7] , and the rapid development of nanoparticles also provides a new idea and direction for cancer treatment [8] . Nanoparticle-mediated targeted therapy is a promising alternative therapy that overcomes drug resistance to a certain extent, enhances the therapeutic effect, and has few side effects [9] . Among various nanoparticles, silver nanoparticles (AgNPs) have been extensively studied.

AgNPs have been used as antibacterial drugs [10, 11] , antiangiogenic agents [12] , and antidiabetic drugs [13] . In addition, AgNPs are cytotoxic to a variety of cancer cells, such as breast cancer [14, 15] , OC [16] , lung cancer [17] , colon cancer [18]  and liver cancer [19] . The toxic mechanisms include increased reactive oxygen species (ROS) production and activation of various apoptosis signalling pathways. Previous studies have shown that AgNPs exert a strong toxic effect on OC cells. Notably, AgNPs synthesized in studies related to OC are all spherical. Because the toxicity of the nanoparticles is related to the shape, this study chemically synthesized triangular silver nanoparticles (tAgNPs) with a particle size of 25–50 nm, and evaluated the anticancer effects of tAgNPs towards human OC SKOV3 cells.

## Materials and methods

### Materials

Silver nitrate (AgNO_3_) and trisodium citrate dihydrate (Na_3_C_6_H_5_O_7_) were purchased from Sinopharm Chemical Reagent Co., Ltd., polyvinylpyrrolidone (PVP, Mw = 29.0 kg·mol^−1^) was purchased from Tianjin Kermel Chemical Reagent Co., Ltd., and sodium borohydride (NaBH_4_, purity 98%) was purchased from Alfa. Human OC SKOV3 cells were obtained from the Cell Bank of Chinese Academy of Sciences (Shanghai, China). Cell Counting Kit-8 (CCK-8) was purchased from Dojindo Molecular Technologies (Dojindo, Japan). The cell cycle, Annexin V-FITC/PI and ROS detection kits were all purchased from Beyotime. Antibodies for western blot (WB) were purchased from Cell Signaling Technology (CST) and Abcam. BALB/c-nu mice were purchased from the Jinan Pengyue Experimental Animal Breeding, Co., Ltd. (Shandong, China).

### Synthesis and characterization of tAgNPs

Five types of AgNPs were synthesized using a chemical reduction method. AgNO_3_ was added to Na_3_C_6_H_5_O_7_, PVP, and H_2_O_2_ with stirring, and stirring was continued for approximately 5 min. An appropriate amount of NaBH_4_ was weighed and directly added to this solution; the solution was placed in a water bath at 27 °C, and stirring was continued for approximately 30 min until the colour of solution changed. The dynamic light scattering (DLS) experiment was carried out with Malvin Laser Particle Sizer (ZEN3690, Malvin company, UK). The synthesized AgNPs were characterized using a scanning electron microscope (SEM, Zeiss, Germany) to obtain SEM images. According to the SEM images, the particle size distribution was plotted using Origin software, and the biological reduction of silver ions at 420 nm was monitored using spectrophotometry.

### Cell viability assay

Human OC SKOV3 cells in the logarithmic growth phase were seeded into 96-well plates at a density of 5 × 10^3^ cells/well and cultured overnight in an incubator (37 °C and 5% CO_2_). Solutions containing different concentrations of tAgNPs (500 ng/ml, 1000 ng/ml, 1500 ng/ml, 2000 ng/ml, 2500 ng/ml, 3000 ng/ml) were prepared with RPMI 1640 complete medium and added into wells at 100 μl/well. A control group and a blank group were also prepared. After 24 h of exposure, the old medium was removed and replaced with 100 μl of new medium containing CCK-8, and the culture was continued for 1–2 h. The optical density (OD) at 450 nm was measured with a microplate reader (BioTek). The cell survival rate was calculated using the following formula:

$$\mathrm{cell\ survival\ rate }(\mathrm{\%}) = ({\mathrm{OD}}_{\mathrm{experimental\ group}} - {\mathrm{OD}}_{\mathrm{blank\ group}})/({\mathrm{OD}}_{\mathrm{control\ group}} - {\mathrm{OD}}_{\mathrm{blank\ group}}) \times 100\mathrm{\%}$$

### Plate colony formation assay

Human OC SKOV3 cells in the logarithmic growth phase were seeded in 6-well plates at a density of 1000 cells/well and cultured overnight in an incubator (37 °C, 5% CO_2_). Complete RPMI 1640 medium was used to prepare a solution of tAgNPs (d = 50 nm) at a concentration of 1000 ng/ml, and a control group was prepared. Cells were cultured in an incubator for 2 weeks (37 °C, 5% CO_2_), and the medium was changed every 3 days. After 2 weeks, the cells were fixed with 4% paraformaldehyde and stained with an appropriate amount of 0.5% crystal violet for 15 min. The excess staining solution was slowly removed with running water, and the cells were air-dried and counted under a microscope (Olympus). The colony formation rate was calculated using the following formula:

$$\mathrm{colony\ formation\ rate }=\mathrm{ number\ of\ clones}/\mathrm{number\ of\ inoculated\ cells }\times 100\mathrm{\%}$$

### Cell cycle experiment

Human OC SKOV3 cells in the logarithmic growth phase were seeded in 6-well plates at a density of 2 × 10^5^ cells/well and cultured overnight in an incubator (37 °C, 5% CO_2_). The culture medium in the 6-well plates was discarded. A solution of tAgNPs (d = 50 nm) at a concentration of 1000 ng/ml was prepared in RPMI 1640 complete medium, and a control group was prepared. Cells were cultured in an incubator for 24 h, 48 h, and 72 h (37 °C, 5% CO_2_). The procedure was performed according to the instructions of the cell cycle detection kit (Beyotime), and detection was performed using a flow cytometer (Beckman Coulter). The data are processed and analyzed by flow JO software (Version 10).

### Cell apoptosis assay

Human OC SKOV3 cells in the logarithmic growth phase were seeded in 6-well plates at a density of 2 × 10^5^ cells/well and cultured overnight in an incubator (37 °C, 5% CO_2_). Complete RPMI 1640 medium was used to prepare a solution of tAgNPs (d = 50 nm) at a concentration of 1000 ng/ml, and a control group was prepared. Cells were cultured in an incubator for 24 h, 48 h, and 72 h (37 °C, 5% CO_2_). The procedure was performed according to the instructions of the Annexin V-FITC/PI apoptosis detection kit (Beyotime), and detection was performed using a flow cytometer (Beckman Coulter). The data are processed and analyzed by CytExpert for DxFLEX software.

### ROS detection

Human SKOV3 OC cells in the logarithmic growth phase were seeded in 6-well plates at a density of 2 × 10^5^ cells/well and cultured overnight in an incubator (37 °C, 5% CO_2_). After 24 h of culture, a solution of tAgNPs (d = 50 nm) at a concentration of 1000 ng/ml was added to the experimental group and cultured for 24 h. Dichloro-dihydro-fluorescein diacetate (DCFH-DA) was diluted with serum-free medium at a ratio of 1:1000, and 1 ml of diluted DCFH-DA was added to each well, followed by an incubation in an incubator for 20 min (37 °C). DCFH-DA was discarded, and the serum-free medium was used to fully wash away the DCFH-DA that did not enter the cells. Cells were observed under an inverted fluorescence microscope (Leica).

### WB

Human OC SKOV3 cells in the logarithmic growth phase that were untreated and treated with 1000 ng/ml tAgNPs were collected, fully lysed and resuspended in sodium dodecyl sulfate polyacrylamide gel electrophoresis (SDS-PAGE) loading buffer. The OD of the extracted protein at 562 nm was determined using the bicinchoninic acid (BCA) protein assay kit (Beyotime), and the protein concentration was calculated. Proteins were transferred to polyvinylidene fluoride (PVDF) membranes after separation on PAGE gels and then incubated with Tris-buffered saline supplemented with Tween (TBST) blocking solution containing 5% nonfat milk for 2 h at room temperature. The membranes were washed with TBST and incubated with antibodies against specific proteins at 4 °C overnight. Rabbit pAb: anti-caspase-3(diluted 1/5000), cyclinA2(diluted 1/2000) and cyclinD1(diluted 1/1000) were used. The membrane was washed three times with TBST and incubated with the secondary antibody (diluted 1/2000) for 1.5 h. TBST was used to remove the unbound secondary antibody, an appropriate amount of chromogenic solution was added to visualize the bands, and images were captured using a luminometer (Tanon).

### In vivo tumorigenesis experiment

BALB/c female nude mice aged 4–5 weeks and weighing 16–18 g were housed in separate cages in a specific-pathogen-free (SPF) animal room, with five animals in each cage. Mice were fed with national standard solid mixed feed, with the free access to water. Human OC SKOV3 cells in the logarithmic growth phase were inoculated subcutaneously into the right thigh of nude mice at a concentration of 2 × 10^7^ cells/ml in a volume of 100 μl per mouse. Mice were randomly divided into Groups A and B, with six mice in each group. Immediately after modelling, mice in Group A were intraperitoneally injected with a solution of tAgNPs (d = 50 nm) at a concentration of 1.513 × 10^4^ ng/ml once every other day in a volume of 300 μl per mouse. In Group B, the injection was started when the tumour grew to approximately 100 mm^3^, and the concentration and dose of tAgNPs (d = 55.7 nm) were the same as those in Group A. The total intervention time was approximately 2 weeks. During the administration period, the mental state, diet and water consumption of the mice were observed daily. The long axis a and short axis b of the tumours in the tumour-bearing mice were measured every 2 days, and the changes in the body weight of the mice were measured. The tumour volume was calculated using the following formula, and the tumour growth and mouse body weight change curves were plotted:

$$\mathrm{V }= 1/2\ {\mathrm{ab}}^{2}$$

### Haematoxylin and eosin (HE) staining

After the nude mice were sacrificed, the heart, liver, spleen, lung, and kidney were removed, washed with normal saline to remove the blood, fixed with 4% paraformaldehyde, processed into paraffin sections for HE staining, and observed under a microscope (Olympus).

### Statistical analysis

All experiments were repeated at least three times. The results are presented as the means ± standard deviations (SD). A T test or one-way analysis of variance (ANOVA) was used to compare all experimental data, and p < 0.05 was considered statistically significant.

## Results

### Synthesis and characterization of tAgNPs

**Fig. 1:**
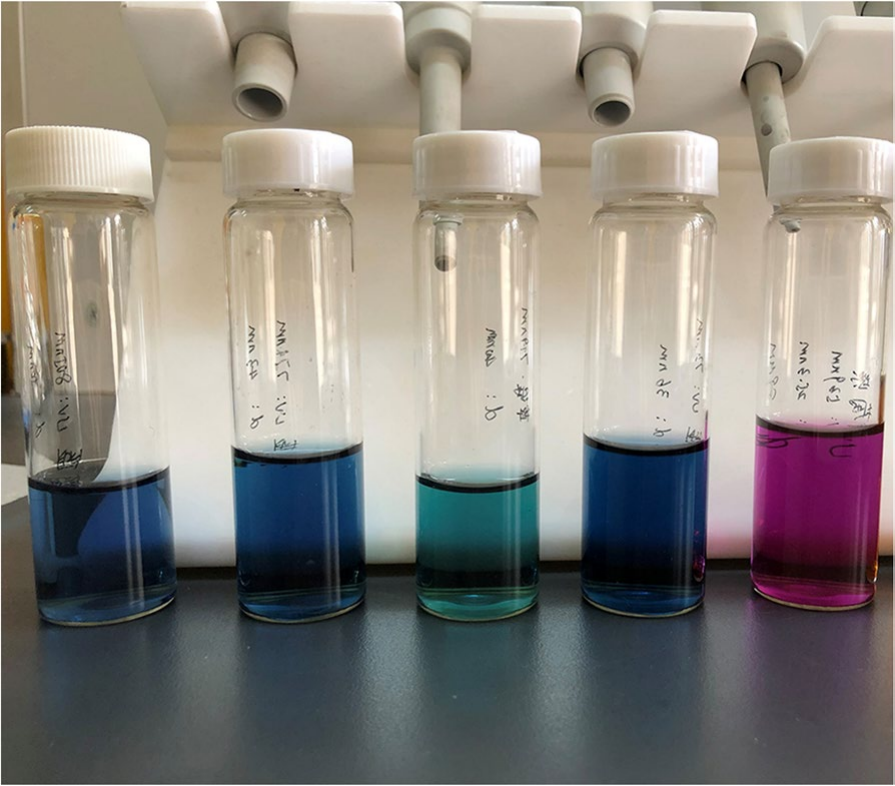
Colour images of the five types of synthesized tAgNPs

**Fig. 2:**
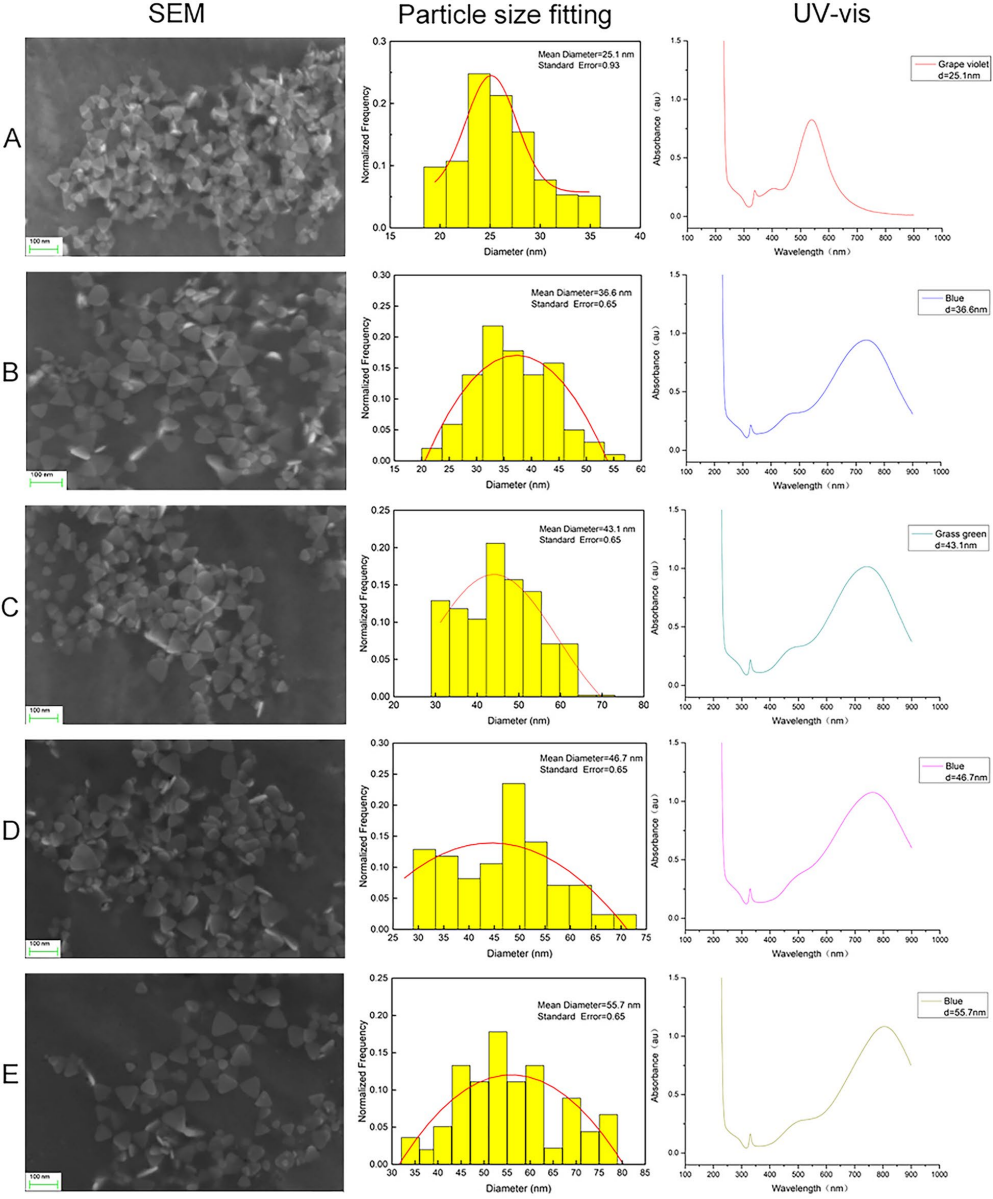
SEM images of the five types of synthesized tAgNPs, the particle size distribution plotted from the SEM images, and the UV–vis spectra at 420 nm. **A**: The particle size of tAgNPs is 25.1 nm and UV peak is 539 nm. **B**: The particle size of tAgNPs is 36.6 nm and the UV peak is 730 nm. **C**: The particle size of tAgNPs is 43.1 nm and the UV peak is 749 nm. **D**: The particle size of tAgNPs is 46.7 nm and the UV peak is 774 nm. **E**: The particle size of tAgNPs is 55.7 nm and the UV peak is 805 nm

The long polymer chain of PVP spirals outward and tightly wraps around the nucleus to form a coating layer, which can prevent the agglomeration of nanoparticles. The preferential adsorption of citrate on different crystal planes of silver nucleus plays a decisive role in the shape of triangular disk. Our SEM images without PVP or citrate proved this (supplementary Fig. 1, supplementary Fig. 2). Therefore, in the synthesis of our tAgNPs, we used PVP and citrate. Five types of tAgNPs with different colours were chemically synthesized (Fig. 1). To better determine the size of the tAgNPs we synthesized, we conducted DLS experiments (supplementary Fig. 3), observed and photographed under SEM, and fitted the SEM image with Origin software (Fig. 2). However, DLS is more accurate for measuring spherical samples, but the test results are not very accurate for samples with irregular shapes such as triangles. Therefore, it is slightly different from the results obtained by software fitting. The SEM images and Origin software fitting results showed that the particle size of the five types of tAgNPs was 25.1 nm, 36.6 nm, 43.1 nm, 46.7 nm, and 55.7 nm. In addition, Ultraviolet–visible (UV–vis) absorption spectroscopy was used to characterize the tAgNPs (Fig. 2). The spectra showed that the five types of tAgNPs have maximum absorption peaks at 539 nm, 730 nm, 749 nm, 774 nm, and 805 nm, respectively.

### Effect of tAgNPs on the viability of human OC cells

**Fig. 3:**
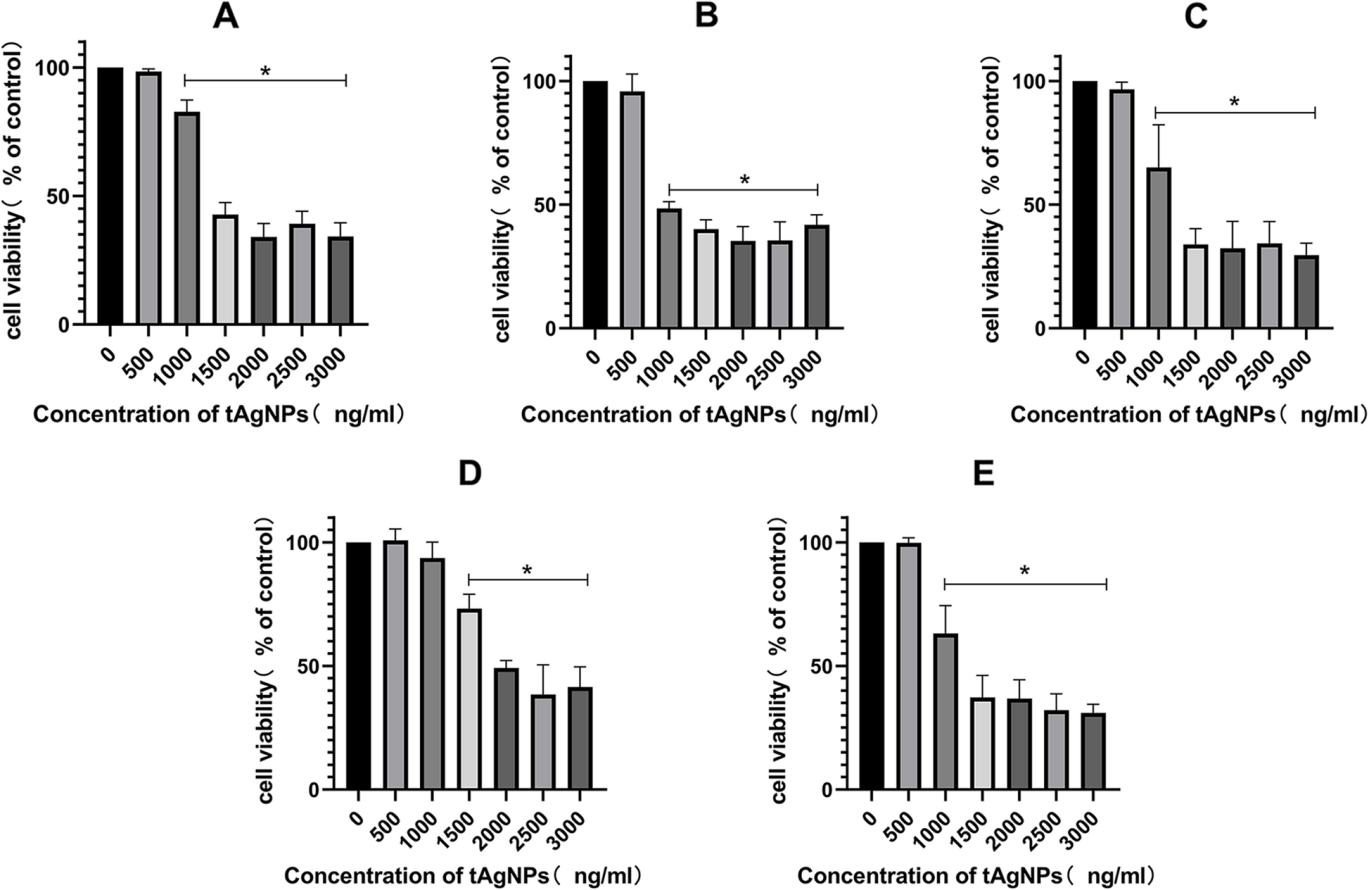
The survival rate of the human OC cell line SKOV3 after 24 h of treatment with different concentrations of tAgNP with different particle diameters was measured using the CCK-8 method. **A** tAgNP diameter: 25.1 nm, UV peak: 539 nm; **B** tAgNP diameter: 36.6 nm, UV peak: 730 nm; **C** tAgNP diameter: 43.1 nm, UV peak: 749 nm; **D** tAgNP diameter: 46.7 nm, UV peak: 774 nm; and E tAgNP diameter: 55.7 nm, UV: 805 nm. The average of three independent repeats is presented as the mean ± SD. The difference between the treatment and control groups was analysed using the T test. The statistical significance of differences between the treatment and control groups is represented by *P < 0.05. Abbreviations: tAgNPs, Triangular silver nanoparticles

The tAgNPs were used to treat the OC cell line SKOV3 at concentrations of 0–3000 ng/ml and particle sizes of 25–56 nm. After 24 h of treatment, the toxic effect of tAgNPs on SKOV3 cells was observed. As shown in Fig. 3, the effects of tAgNPs with different particle sizes ranging from 25–56 nm on cell viability were approximately the same, with no significant differences, and tAgNPs reduced the growth and viability of human OC cells in a dose-dependent manner, consistent with the findings reported by Gurunathan et al. [17] in lung cancer. After the cells were treated with tAgNPs for 24 h, AgNPs at a concentration of 1000 ng/ml or higher exhibited significant cytotoxicity towards the cells. Therefore, tAgNPs with a particle size of 55.7 nm and a concentration of 1000 ng/ml were used in subsequent experiments.

### Inhibitory effect revealed using the plate colony formation assay

**Fig. 4:**
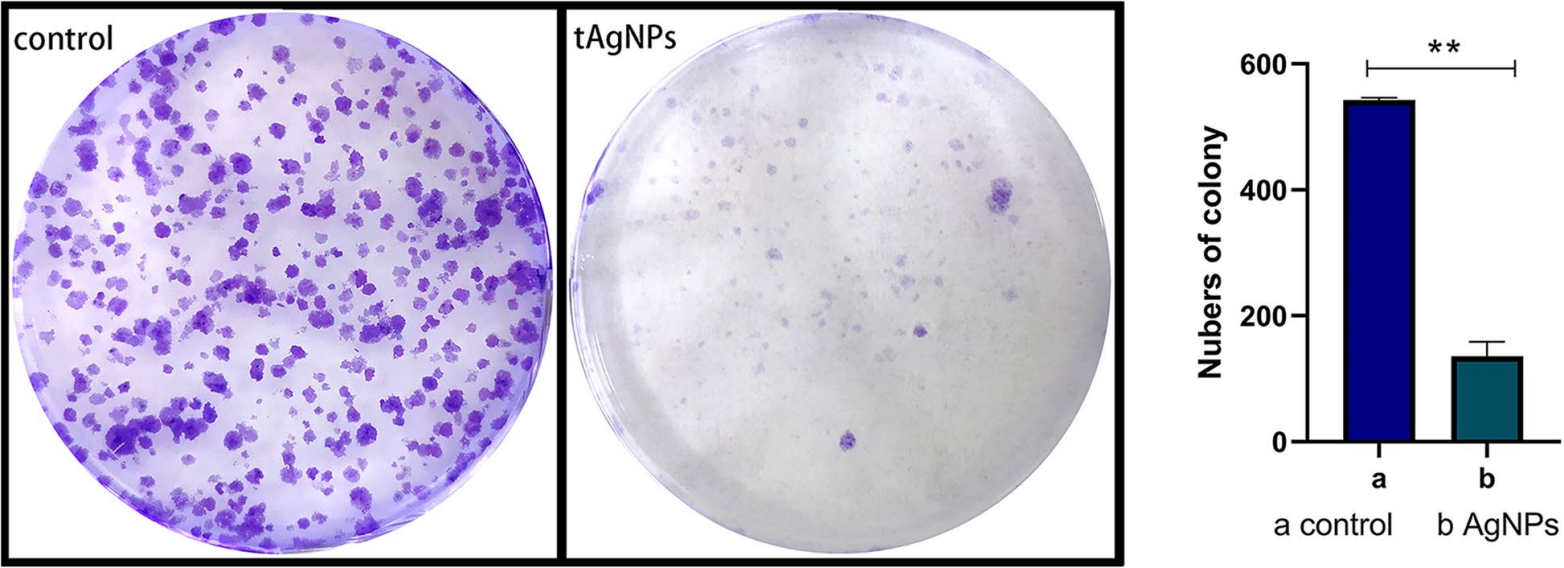
The image in the left panel shows the crystal violet-stained control and treatment groups after 2 weeks of culture. The right panel shows the quantitative analysis of the inhibitory effect on colony formation. The average of three independent repeats is presented as the mean ± SD. The difference between the treatment and control groups was analysed using the T test. The statistical significance of the difference between the treatment and control groups is represented by **P < 0.01

SKOV3 cells were prepared as a single-cell suspension and inoculated in 6-well plates at a density of 1000 cells/well. After 2 weeks of culture with 1000 ng/ml tAgNPs, cells were stained with crystal violet to observe the inhibitory effect of tAgNPs on cell proliferation. As shown in Fig. 4, compared with SKOV3 cells that were not treated with tAgNPs, SKOV3 cells treated with tAgNPs had a significantly lower colony formation ability. Subsequently, the inhibitory effect of tAgNPs on SKOV3 cells was quantitatively analysed by counting cells using ImageJ software. The clone formation rate of the control group was 54.25%, and the clone formation rate of the experimental group was 13.55%.

### Effect of tAgNPs on cell cycle

**Fig. 5:**
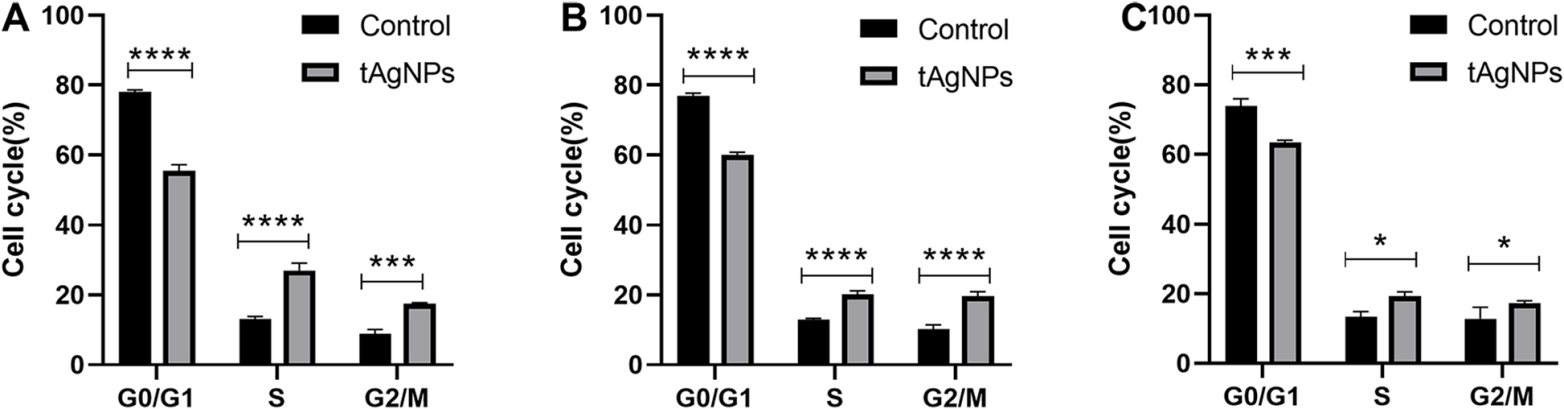
Cell cycle distribution of OC SKOV3 cells treated with tAgNPs for 24, 48, and 72 h and untreated cells. The average of three independent repeats is presented as the mean ± SD. The difference between the treatment and control groups was analysed using the T test. The statistical significance of the difference the treatment and control groups is represented by *P < 0.05, ***P < 0.001, and ****P < 0.0001

**Fig. 6:**
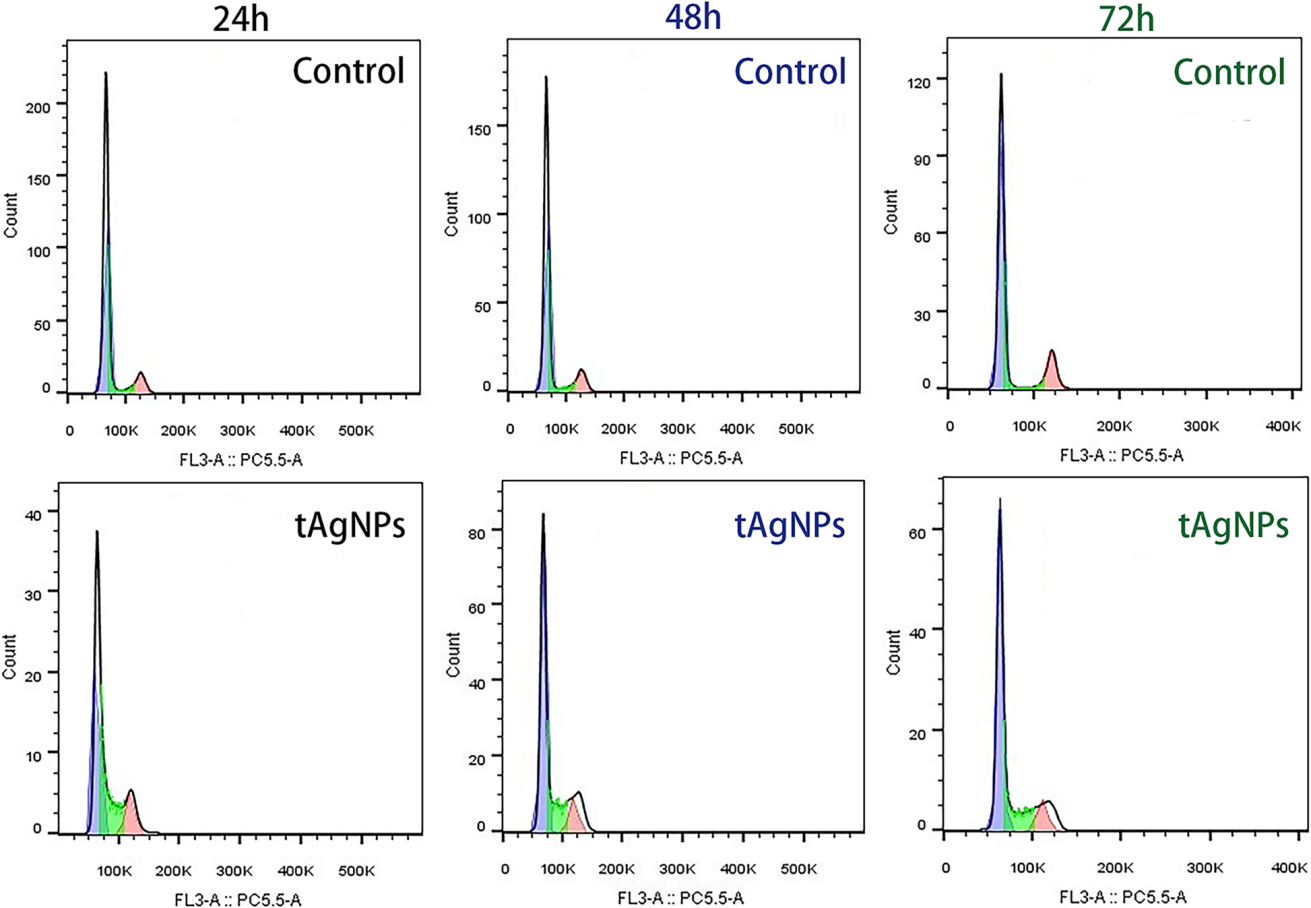
After the OC SKOV3 cells treated with 1000 ng/ml tAgNPs for 24, 48, and 72 h, the flow cytometry results were plotted using Flow Jo software

Untreated SKOV3 cells were used as the control group, and SKOV3 cells were treated with tAgNPs for 24 h, 48 h, and 72 h to observe the effect of tAgNPs on the cell cycle of OC cells. Changes in the cell cycle of SKOV3 cells were quantitatively detect using a cell cycle detection kit and a flow cytometer. We made the following analysis (Table 1, Fig. 5, Fig. 6), Over time, the number of untreated SKOV3 cells in G0/G1 phase decreased slightly, the number in S phase did not change significantly, and the number in G2/M phase increased. For the tAgNP-treated cells, the number in G0/G1 phase increased and the number in S phase decreased in a time-dependent manner. The change observed between 48 and 72 h was not significant, while that between 24 and 48 h was significant.

**Table 1:** The results of cell cycle distribution of SKOV3 cells

Time	Treatment	Cell cycle		
		G0/G1	S	G2/M
24 h	Control	78.12 ± 0.48	13.02 ± 0.83	8.87 ± 1.27
	tAgNPs	55.47 ± 1.74	27.07 ± 2.03	17.47 ± 0.35
48 h	Control	76.88 ± 0.84	12.87 ± 0.42	10.27 ± 1.17
	tAgNPs	60.03 ± 0.83	20.23 ± 0.85	19.74 ± 0.02
72 h	Control	73.84 ± 2.09	13.37 ± 1.50	12.80 ± 3.31
	tAgNPs	63.40 ± 0.67	19.34 ± 1.18	17.27 ± 0.68

The results are presented as the means ± SD. (*n* = 3)

### Effect of tAgNPs on cell apoptosis

**Fig. 7:**
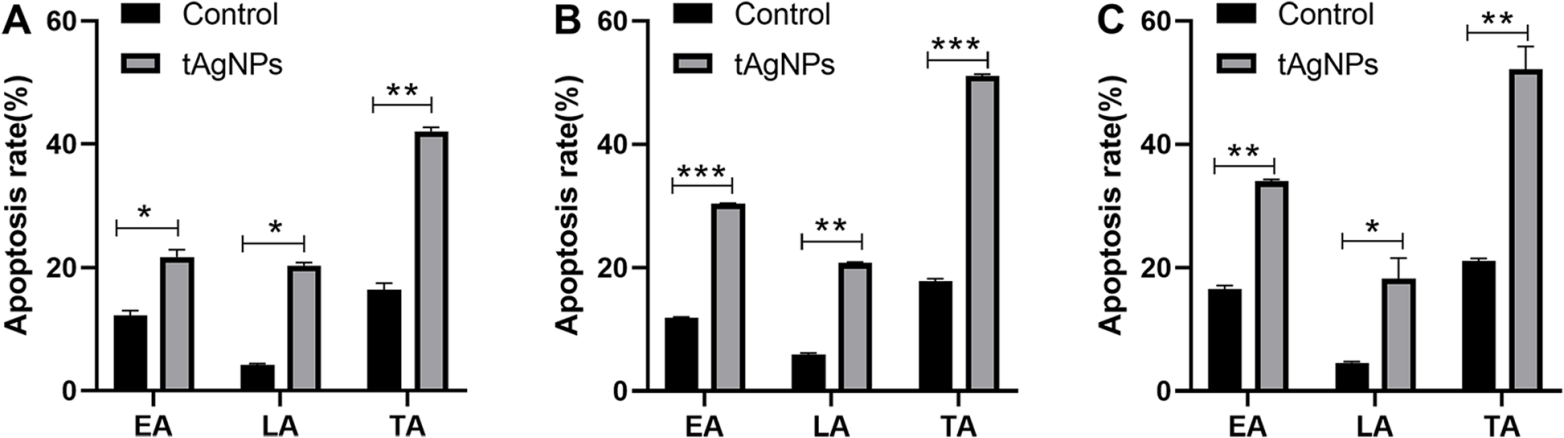
Distributions of apoptotic human OC SKOV3 cells treated with tAgNPs for 24, 48, and 72 h and untreated cells. The average of three independent repeats is presented as the mean ± SD. The difference between the treatment and control groups was analysed using the T test. The statistical significance of the difference the treatment and control groups is represented by *P < 0.05, **P < 0.001, and ***P < 0.0001. Abbreviations: EA, Early apoptotic cells, LA, Late apoptotic cells, TA, Total apoptotic cells

**Fig. 8:**
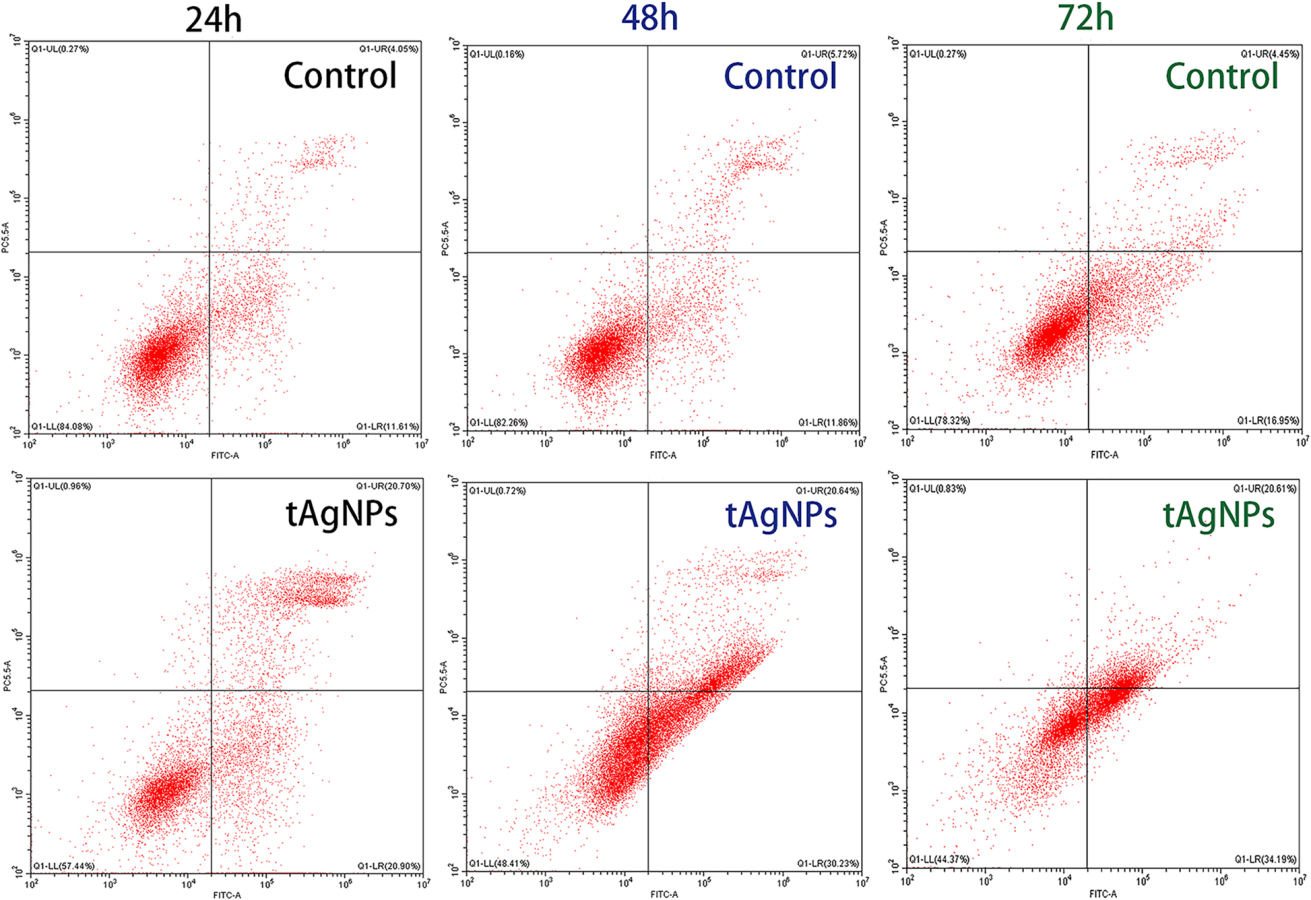
Flow cytometry results plotted for human OC SKOV3 cells treated with 1000 ng/ml tAgNPs for 24, 48, and 72 h

An Annexin V-FITC apoptosis detection kit was used to determine the apoptosis rate. Annexin V has a high affinity for phosphatidylserine on the cell membrane surface and is used to label apoptotic cells; PI is used to determine the integrity of the cell membrane and detect necrotic cells. After gating using flow cytometry, the quadrants were located on the Annexin V/PI dot plot to distinguish living cells, early apoptotic cells, late apoptotic cells, and necrotic cells. According to the experimental results (Table 2, Fig. 7, Fig. 8), the total apoptosis rate of human OC SKOV3 cells treated with tAgNPs at 24, 48, and 72 h was 42.08 ± 0.67%, 51.08 ± 0.30%, and 52.20 ± 3.68%, respectively. The tAgNPs significantly increased cell apoptosis in a time-dependent manner, as little change was observed between 48 and 72 h.

**Table 2:** The results of cell apoptosis of SKOV3 cells

Time	Treatment	Cell distribution(%)			
		Live cells	Early apoptotic cells	Late apoptotic cells	Total apoptotic cells
24 h	Control	83.52 ± 0.81	12.21 ± 0.84	4.20 ± 0.21	16.41 ± 1.05
	tAgNPs	57.13 ± 0.44	21.73 ± 1.17	20.35 ± 0.50	42.08 ± 0.67
48 h	Control	82.00 ± 0.37	11.93 ± 0.10	5.91 ± 0.27	17.84 ± 0.37
	tAgNPs	48.20 ± 0.30	30.32 ± 0.13	20.76 ± 0.17	51.08 ± 0.30
72 h	Control	78.62 ± 0.42	16.56 ± 0.56	4.58 ± 0.18	21.14 ± 0.37
	tAgNPs	47.04 ± 3.78	33.97 ± 0.31	18.23 ± 3.37	52.20 ± 3.68

The results are presented as the means ± SD. (*n* = 3)

### Effect of tAgNPs on increasing ROS production

**Fig. 9:**
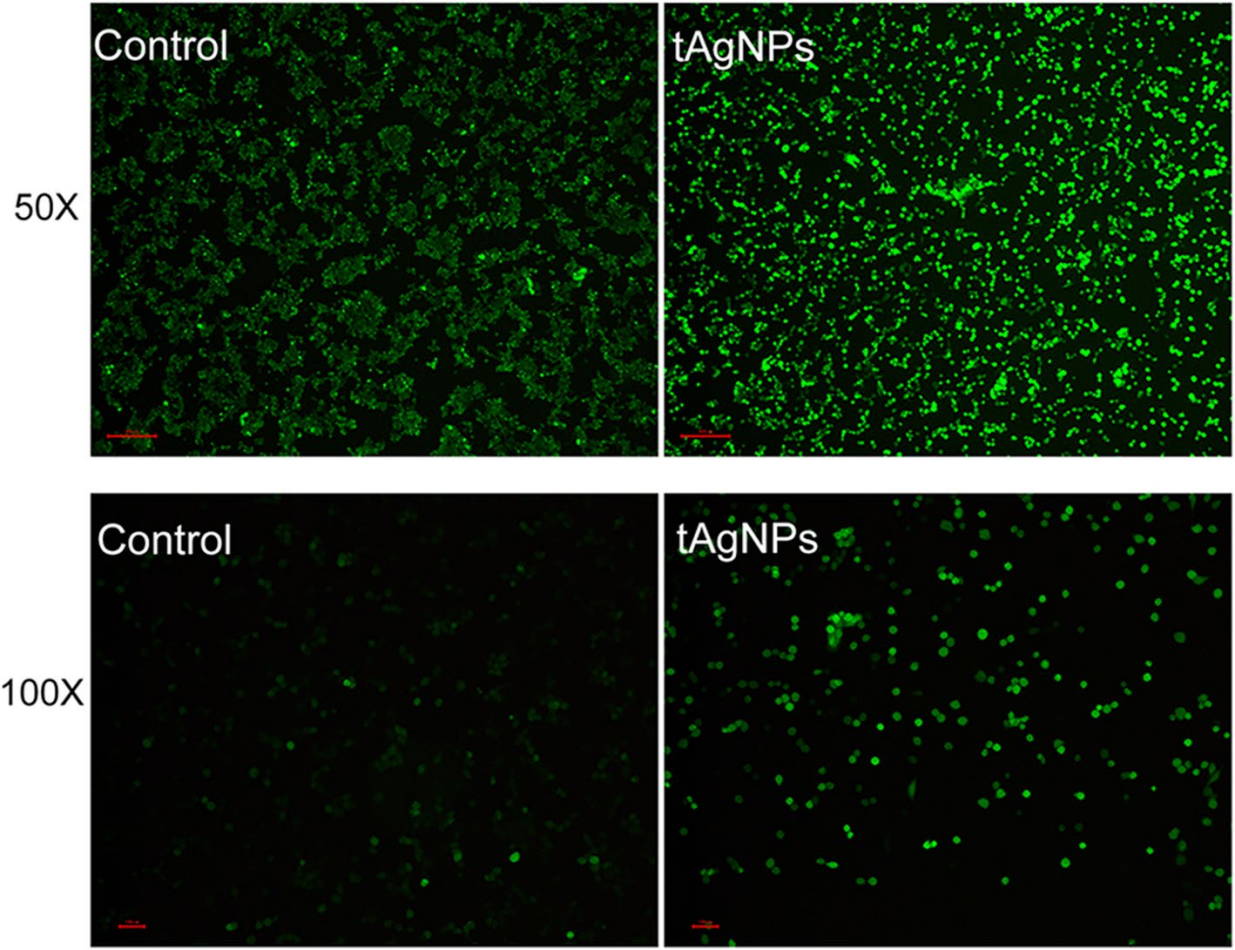
Images of SKOV3 cells treated with tAgNPs for 24 h and untreated cells under an inverted fluorescence microscope at 50X and 100X magnification

Relevant studies have reported that AgNPs exposure may increase intracellular ROS levels [20]. Therefore, the effect of tAgNPs on ROS level was investigated. After SKOV3 cells were treated with tAgNPs for 24 h, the oxidative stress indicator DCFH-DA was used to evaluate the intracellular ROS level. Under an inverted fluorescence microscope, SKOV3 cells treated with 1000 ng/ml tAgNPs showed strong green fluorescence, indicating a high ROS level, as shown in Fig. 9.

### WB mechanisms of cell growth inhibition by tAgNPs

**Fig. 10:**
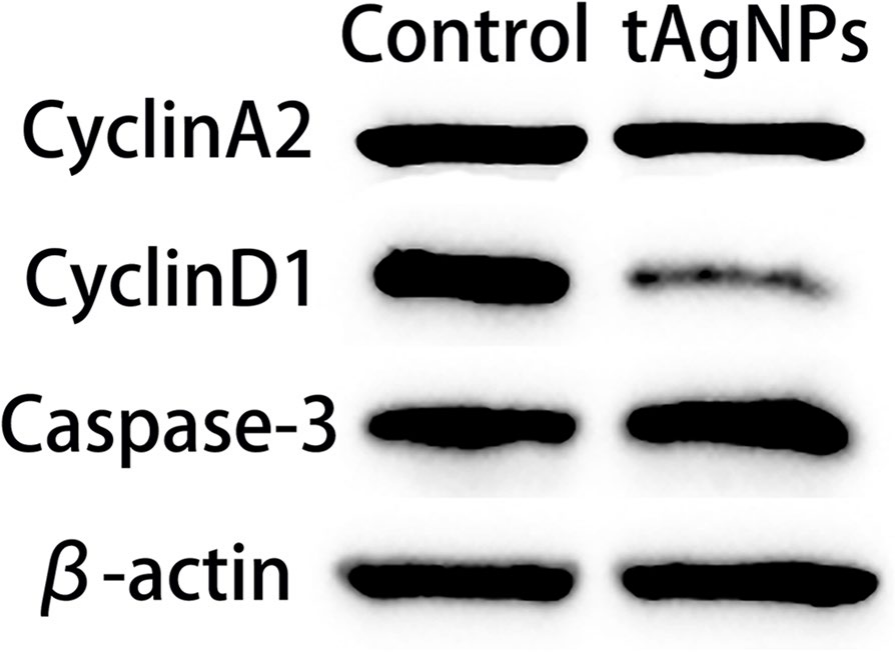
Effect of tAgNPs on the expression levels of apoptosis- and proliferation-related proteins in SKOV3 cells

Subsequently, we aimed to further understand the effect of tAgNPs treatment on the expression levels of proliferation- and apoptosis-related proteins in OC cells. WB was performed to analyse the levels of proliferation-related proteins, such as the transcription factor cyclinA2, cyclinD1, and the apoptosis-related protein caspase-3 in SKOV3 cells exposed to 1000 ng/ml tAgNPs and untreated SKOV3 cells. As shown in Fig. 10, the expression levels of cyclinA2, and cyclinD1 decreased, caspase-3 expression increased, and the expression of β-actin was unchanged in SKOV3 cells treated with tAgNPs compared to untreated SKOV3 cells.

### In vivo antitumor effect research

**Fig. 11:**
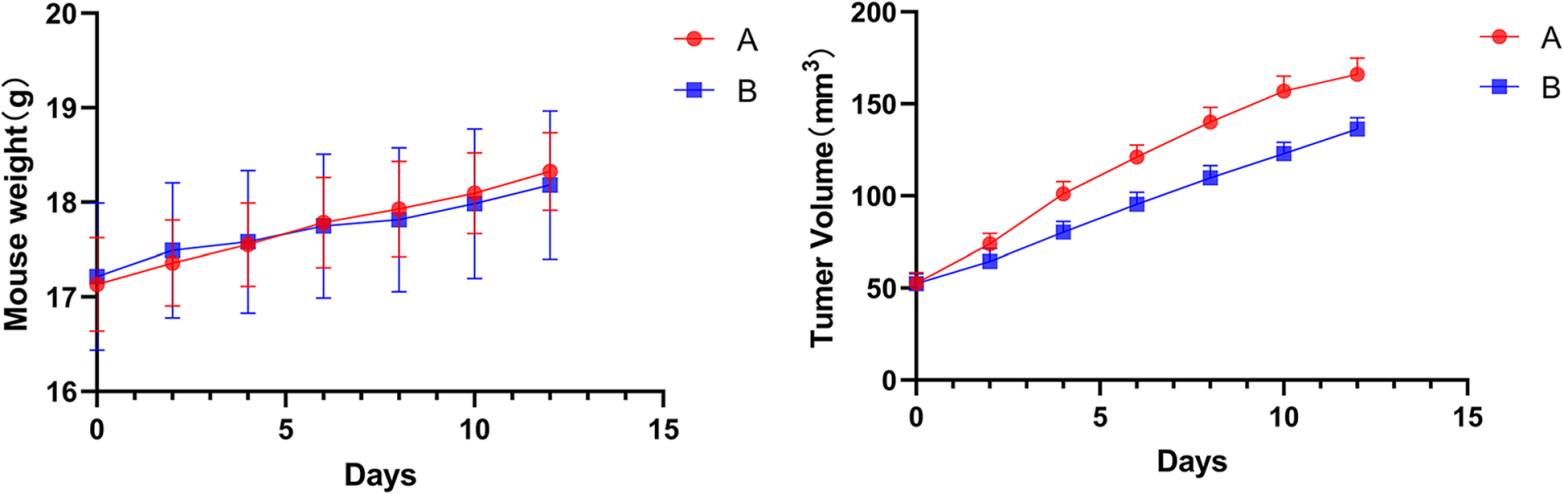
Changes in the body weight and tumour volume of the two groups of nude mice during the intervention period. **A**: Treatment with tAgNPs when the tumour volume reached approximately 100 mm^3^. **B**: Treatment with tAgNPs at the beginning of tumour formation by subcutaneous injection. The results are presented as the means ± SD

**Fig. 12:**
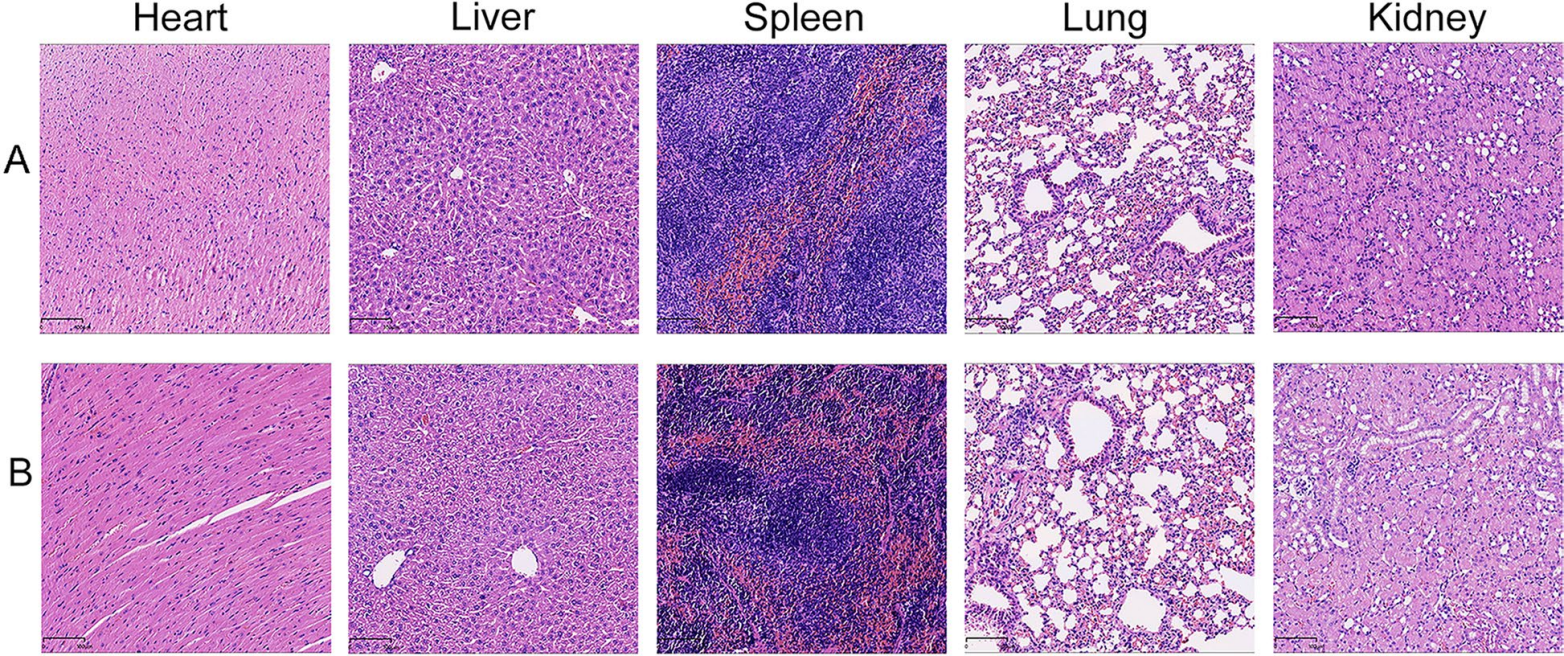
Microscopic images (100X) of the main organs of the two groups of nude mice bearing SKOV3 tumours after 14 days of the intervention. **A**: Treatment with tAgNPs when the tumour volume reached approximately 100 mm^3^. **B**: Treatment with tAgNPs at the beginning of tumour formation by subcutaneous injection

SKOV3 cells were subcutaneously injected into Balb/c nude mice to induce tumour formation and determine whether tAgNPs are useful for treatment in vivo. One group was treated with tAgNPs from the beginning of tumour formation, and the other group was treated with tAgNPs when the tumour grew to approximately 100 mm^3^. During treatment, the weight, mental state and tumour volume of the nude mice were closely observed. As shown in Fig. 11, no significant difference in body weight was observed between the two groups of nude mice, but the tumour volume of the nude mice treated with tAgNPs at the beginning increased more slowly than that in Group A. During the intervention period, the nude mice in both groups were in good spirits and able to move freely. After 2 weeks of the intervention, the nude mice were sacrificed, and the heart, liver, spleen, lung, and kidney were collected for HE staining. As shown in Fig. 12, the heart, liver, spleen, lung and kidney of the two groups were basically the same after HE staining, and no significant changes were observed, indicating that tAgNPs did not cause abnormalities in normal organs in vivo.

## Discussion

According to previous studies, AgNPs have been rapidly developed due to their antibacterial and anticancer effects. However, the cytotoxicity of AgNPs depends on many factors, such as the exposed cell type and the size and shape of the nanoparticles [21] . We noticed that the role of the shape of AgNPs in cancer treatment has not been explored. Therefore, in the present study, five types of tAgNPs with different sizes were synthesized and characterized to explore their role in OC treatment. The diameter and morphology of the five types of tAgNPs were analysed using SEM and Origin software. The SEM images showed that AgNPs were all well-dispersed and uniform triangular particles (Fig. 2). Before investigating the effect of tAgNPs on OC cells, a cell viability assay was first performed to detect the cytotoxic effect of tAgNPs with different particle sizes on SKOV3 cells. The results showed (Fig. 3) that OC cells treated with tAgNPs with different particle sizes exhibited significantly reduced proliferation in a dose-dependent manner, but no significant differences were observed between tAgNPs with particle sizes ranging from 25–56 nm. Therefore, this study randomly selected the 55.7 nm tAgNPs for subsequent experiments. We obtained more data and confirmed that tAgNPs impaired the colony-forming ability of SKOV3 cells by performing plate colony formation assay and verified the significant inhibitory and toxic effects of tAgNPs on the proliferation of OC cells. In addition, the cell cycle distribution after 24, 48, and 72 h of tAgNP treatment was analysed using flow cytometry. The cytotoxic effect of tAgNPs blocked the transition of cells from G0/G1 phase to S phase, and thus the cells were arrested in G0/G1 phase. However, there was little change in cell cycle from 48 to 72 h, which may be related to the time dependence of tAgNPs.

Cell apoptosis is a basic and complex physiological process and an adaptive response that is involved in development, differentiation, and homeostasis, and limits the survival and development of malignant cells. Cancer cells often undergo mutation in a certain link in the programmed cell death pathway to avoid apoptosis. At present, most studies have attempted to affect drug resistance during chemotherapy by activating the cell death pathway in cancer cells, thereby affecting the outcome of cancer treatment. Therefore, many anticancer drugs or chemotherapeutic drugs exert a therapeutic effect by enhancing cell apoptosis [22–24] . In this study, cell apoptosis was quantitatively analysed using Annexin V-FITC/PI staining and flow cytometry (Figs. 7 and 8), and the results confirmed that tAgNPs treatment induced apoptosis in SKOV3 OC cells. Similar to the results of cell cycle experiment, tAgNPs showed similar apoptotic effects at 48 h and 72 h, which once again confirmed that tAgNPs may have time-dependent characteristics. The cell apoptosis process involves various signalling pathways. Relevant studies have shown that ROS and cysteine proteases are involved in the apoptosis pathways [25] . This study first verified whether tAgNP-induced apoptosis in OC cells is related to ROS production. ROS plays an important role in various physiological and pathological processes, and a normal physiological ROS level is involved in the initiation of apoptosis signalling pathways [26]. In the present study, the ROS level in SKOV3 cells was detected using the DCFH-DA assay and was significantly increased in OC cells treated with tAgNPs. ROS exert a cytotoxic effect on cells [20, 27] . Oxidative stress caused by increased ROS production leads to dysfunction of the cellular antioxidant system [26] , which may cause the death of cells treated with tAgNPs. The tAgNPs treatment increased the ROS level in cells, reduced the antioxidant capacity of cells, and caused oxidative damage in cells, leading to apoptosis, consistent with the previously reported cytotoxicity of AgNPs towards human breast cancer and lung cancer [28, 29] , and indicating that ROS production plays a key role in tAgNP-induced cytotoxicity.

Cysteine proteases are the central link in the process of cell apoptosis. For example, the Bcl-2 family members balance each other to regulate the release of cytochrome c [30–32] and activate cysteine proteases, such as caspase-3 and caspase-9 [33–36] , leading to cell apoptosis. We investigated the effect of tAgNPs treatment on caspase-3 levels to determine whether cell apoptosis was activated through the cysteine protease-mediated pathway. The WB results showed increased caspase-3 expression in the cells treated with tAgNPs. Relevant studies have shown that AgNPs activate caspase-3 and promote cell apoptosis, thereby inhibiting tumour growth [37, 38] . Studies have confirmed that AgNPs increase the caspase-3 level in breast cancer [39], lung cancer [40]  and other cancers, indicating that tAgNP-treated OC cells complete the apoptosis process through the cysteine protease pathway.

In addition, this study examined the levels of several factors related to proliferation, such as cyclinA2, and cyclinD1, which are key proteins that regulate the cell cycle and proliferation. Some studies have shown that certain drugs inhibit tumour proliferation by downregulating the expression of the cell cycle proteins cyclinA2 and cyclinD1, thereby exerting an anticancer effect [41, 42] . In the present study, the expression levels of cyclinA2, and cyclinD1 were reduced in OC cells treated with tAgNPs, indicating that tAgNPs may delay tumour progression by inhibiting cell proliferation.

As a method to further understand the toxic side effects of tAgNPs in vivo, OC cells were injected into female nude mice to cause subcutaneous tumour formation. After the intervention, tAgNPs delayed the growth of tumours, indicating that tAgNPs exert antitumor effect on OC. In addition, during the entire experiment, no significant differences in body weight were observed between the two groups of SKOV3 OC-bearing mice receiving different treatments (Fig. 11A), and HE staining of important organs showed no significant changes (Fig. 12), indicating that tAgNPs may not be toxic to normal organs. Therefore, the dose adopted in this study exerts a certain antitumour effect without toxic side effects, indicating that tAgNPs have the potential for further clinical application in the treatment of OC.

## Conclusions

The purpose of this study was to determine the effect of the tAgNPs on the treatment of OC cells. This study chemically synthesized five types of tAgNPs with particle sizes of 25–56 nm. The properties of all tAgNPs were stable. The results of the cytotoxicity experiment showed that tAgNPs could reduce the survival rate of the cells, and there no significant differences among different types. Therefore, tAgNPs with a particle size of 50 nm were randomly selected for subsequent experiments. tAgNPs significantly inhibited the viability of SKOV3 cells by promoting ROS production, activating the cysteine protease-mediated apoptosis pathway, and inhibiting the expression of proliferation-related factors. In vivo experiments also showed that tAgNPs exerted antitumour effects on OC and had no obvious toxic side effects on normal organs, indicating that tAgNPs are safe for anticancer treatment to a certain extent. This study helps to expand the application of AgNPs in targeted therapy for OC.

## Supplementary Information

**Additional file 1**: **Supplementary figure 1**. SEM image of nanomaterial synthesized without PVP.

**Additional file 2**: **Supplementary figure 2**. SEM image of nanomaterial synthesized without citrate.

**Additional file 3**: **Supplementary figure 3**. DLS results of the five types of synthesized tAgNPs were analyzed by Origin software.

## Data Availability

All data generated or analyzed during this study are included in this published article.

## Abbreviations

AgNPs: Silver nanoparticles
OC: Ovarian cancer
tAgNPs: Triangular silver nanoparticles
SEM: Scanning electron microscopy
UV: Ultraviolet
CCK-8: Cell counting kit-8
ROS: Reactive oxygen species
WB: Western blot
AgNO_3_: Silver nitrate
Na_3_C_6_H_5_O_7_: Trisodium citrate dehydrate
PVP: Polyvinylpyrrolidone
NaBH_4_: Sodium borohydride
DLS: Dynamic light scattering
OD: Optical density
DCFH-DA: Dichloro-dihydro-fluorescein diacetate
SDS-PAGE: Sodium dodecyl sulfate polyacrylamide gel electrophoresis
BCA: Bicinchoninic acid
PVDF: Polyvinylidene fluoride
TBST: Tris-buffered saline supplemented with Tween
SPF: Specific-pathogen-free
UV–vis: Ultraviolet–visible
EA: Early apoptotic cells
LA: Late apoptotic cells
TA: Total apoptotic cells
