# Supplementary figures and images for: Preparation of triangular silver nanoparticles and their biological effects in the treatment of ovarian cancer
Source: J Ovarian Res. 2022 Nov 21;15:121. doi: 10.1186/s13048-022-01056-3 (PMC9680130; doi:10.1186/s13048-022-01056-3)

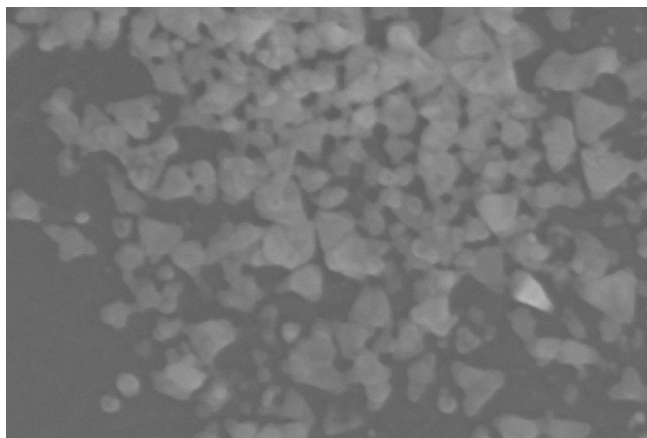

Supplementary figure 1: SEM image of nanomaterial synthesized without PVP.

Supplement: Supplementary file 1 — Additional file 1: Supplementary figure 1. SEM image of nanomaterial synthesized without PVP. [file 13048_2022_1056_MOESM1_ESM.pdf]

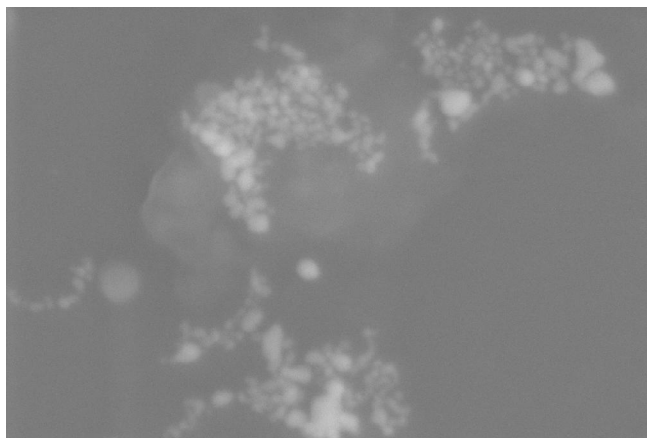

Supplementary figure 2: SEM image of nanomaterial synthesized without citrate.

Supplement: Supplementary file 2 — Additional file 2: Supplementary figure 2. SEM image of nanomaterial synthesized without citrate. [file 13048_2022_1056_MOESM2_ESM.pdf]
