# Supplementary material for: Preparation of triangular silver nanoparticles and their biological effects in the treatment of ovarian cancer
Source: J Ovarian Res. 2022 Nov 21;15:121. doi: 10.1186/s13048-022-01056-3 (PMC9680130; doi:10.1186/s13048-022-01056-3)
Supplement: Supplementary file 3 — Additional file 3: Supplementary figure 3. DLS results of the five types of synthesized tAgNPs were analyzed by Origin software. [file 13048_2022_1056_MOESM3_ESM.pdf]

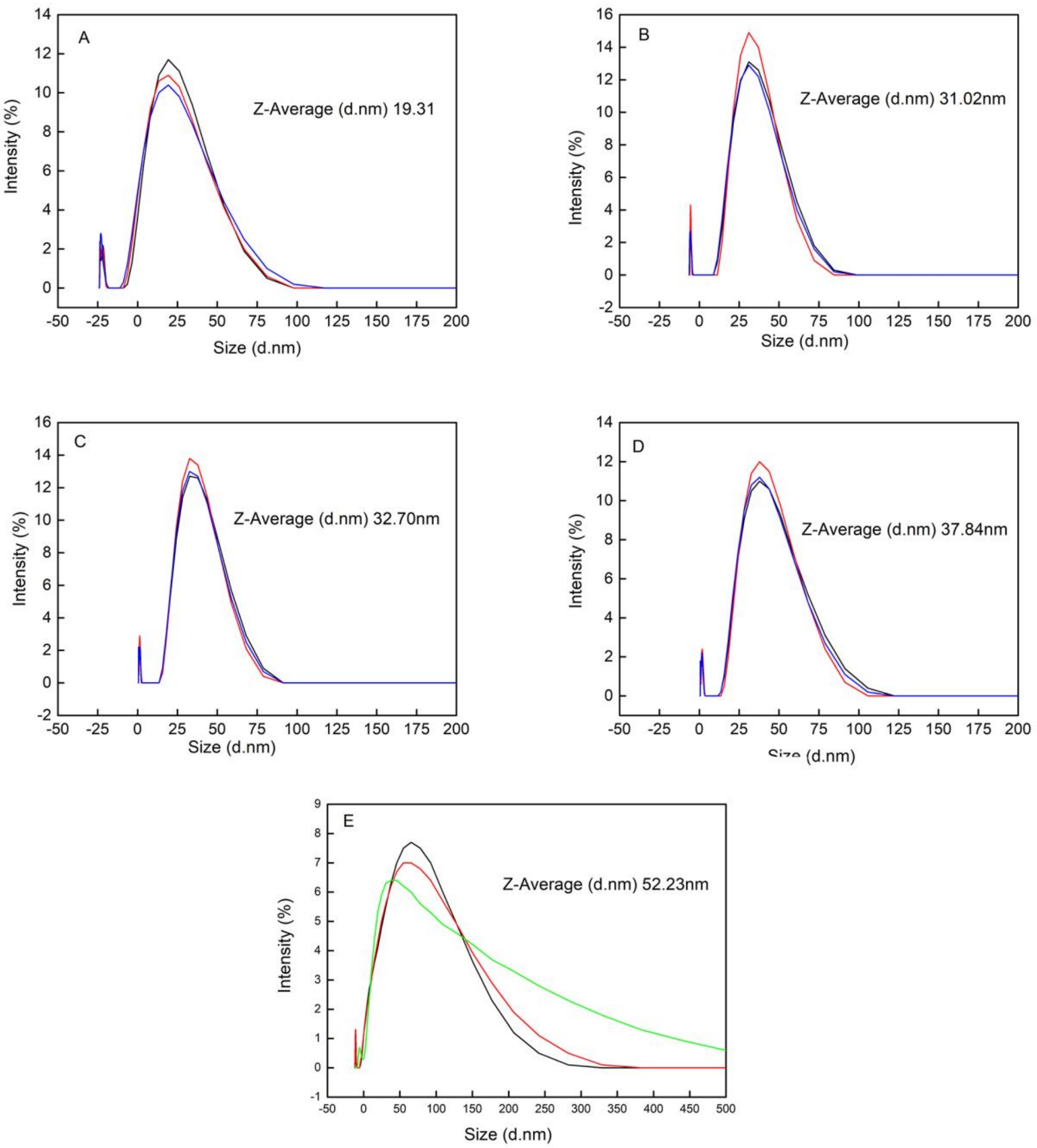

Supplementary figure 3: DLS results of the five types of synthesized tAgNPs were analyzed by Origin software.
